# Supplementary material for: CRISPR-Cas9 Gene Editing and Secondary Metabolite Screening Confirm Fusarium graminearum C16 Biosynthetic Gene Cluster Products as Decalin-Containing Diterpenoid Pyrones
Source: J Fungi (Basel). 2023 Jun 23;9(7):695. doi: 10.3390/jof9070695 (PMC10381663; doi:10.3390/jof9070695)
Supplement: Supplementary file 1 [file jof-09-00695-s001.zip › jof-2434149-supplementary.pdf]

CRISPR-Cas9 gene editing and secondary metabolite screening confirm *Fusarium graminearum* C16 biosynthetic gene cluster products as decalin-containing diterpenoid pyrones.

Carmen Hicks[1,2], Thomas E. Witte[1], Amanda Sproule[1], Anne Hermans[1], Samuel W. Shields[1], Ronan Colquhoun[1], Chris Blackman[1], Christopher N. Boddy[2], Rajagopal Subramaniam[1], David P. Overy[1]

SUPPLEMENTARY INFORMATION

Detailed Metabolomics Workflow: Data Pre-Processing

UPLC-HRMS profiles of both broth and mycelium extracts were processed together, while UPLC-HRMS profiles of the in planta extracts were processed separately; all data were compiled into a representative matrix of metabolite mass features denoted with a retention time (RT) and mass to charge ratio (*m/z*). Raw data files included MeOH blanks run after every sixth sample, and all were carefully examined to determine a minimum noise level threshold for data analysis. Processing of the UPLC-HRMS raw data files was completed using MzMine 2.53 (Pluskal et al., 2010). Mass features were detected with an exact mass noise threshold of 1E4 and chromatographic building was completed with the ADAP algorithm using a minimum group size of 5, a group intensity threshold of 5E6 and a minimum highest intensity of 1E7 for the in vitro experiment, and a group intensity threshold of 1E5 and a minimum highest intensity of 2E5 for the in planta experiment. RT and *m/z* tolerances were consistently set to 0.01 min and 0.001 m/z or 5 ppm throughout all phases of processing. Smoothing was then applied at a filter width of 5. Deconvolution of the chromatograms was completed using the wavelets ADAP algorithm with a S/N of 1, minimum feature height of 1E4, coefficient area of 110, a peak duration range of 0.0-10.00 min and an RT wavelet range of 0.0-0.1 min. Following deconvolution, isotopic peaks were grouped together, and the JOIN aligner was used using a 20:10 *m/z* to RT weight. Same RT and *m/z* gap filling was then applied to back call in any points that were excluded from the original processing parameters but present in the data set.

Metabolomics: Binary Transformation and Data Reduction

Data matrices of mass feature peak height intensities from the in vitro WT media experiments were imported into R Studio for data processing (R Core Team, 2022). Maximum peak height intensities from MeOH blanks for each mass feature were back subtracted from all sample masses in order to reduce false positives resulting from sample carry-over and/or gap filling errors. All mass feature values transformed into a binary presence absence matrix in which values >1E4 were converted to 1, and values <1E4 converted to 0 (following protocols by Witte & Overy, 2021). Mass feature occurrences of individual sample replicates were then consolidated by applying a 75% inclusion (3/4 replicates) on mass features (i.e. a feature had to be >1E4 in a minimum of 3 samples to be considered detected within a sample) to generate a matrix containing 304 mass features. The final media consensus matrix was generating, compressing all sample types per medium, using the sums from binary consensus for each of the media types to obtain an overview of all potential features detected per media type across the various conditions. Consensus heatmaps were generated using the final media consensus as a guide for the clustering algorithm. The “pheatmap” R package, was used to generate the initial heatmap with the ‘ward.D2’ applied as both row and column clustering algorithm (Kolde R, 2019). This clustering pattern

was then applied to each of the four extract/culture type binary consensus' in order to sort all features and samples to correspond with the media consensus format.

Metabolomics: Statistical Analysis

For data comparison of the WT and gene knock out mutants extract profiles from the Q6 media study and the *in planta* infection trials, the resulting data matrix was exported from MZmine as a csv file as a matrix of peak height intensities and imported into R Studio for further processing. First, the maximum detected blank value for each mass feature was back subtracted from all sample masses in order to reduce false positives that may result from either sample carry-over or gap filling errors. The data was then normalized to the total ion current by sample sums. Univariate analysis was completed using the 'MUMA' R package which utilizes both Welch's T tests and Wilcoxon-Mann Whitney U tests for normally and non-normally distribution mass features respectively based on Shapiro scores when determining P-values (Gaude et al., 2013). This is advantageous as mass features constructed or containing potions constructed by knocked out genes are expected to be absent from KO strains, thereby deviating from normal distribution. Mass feature P-value comparing the ΔPKS15 and WT strain were then used to generate plots against their respective retention time. To further examine the features of statistical significance, features with P-values <0.05 were plotted by RT and *m/z*. XICs of mass features were clipped from Xcalibur with a ±5 ppm tolerance on the predicted exact mass.

Media and Reagent Formulations Used: Generation of CRISPR Transformants

CMC Medium

Carboxymethylcellulose	15g
NH_4NO_3	1g
KH_2PO_4	1g
$MgSO_4 \cdot 7H_2O$	0.5g
Yeast Extract	1g
MilliQ Water	to 1.0L

Littman Oxgall Medium

Oxgall powder	15.0g
Dextrose	10.0g
Peptone	10.0g
MilliQ Water	to 1.0L

Note: Littman Oxgall medium has been observed to promote hyphal branching in actively growing mycelia, likely aiding in the formation of protoplasts

Liquid TB3 Medium

Yeast Extract	3.0g
Casamino Acids	3.0g
Sucrose	200.0g
MilliQ Water	to 1.0L

Molten TB3 Medium

Yeast Extract	3.0g
Casamino Acids	3.0g

Sucrose	200.0g
LMP Agarose	7.0g
MilliQ Water	to 1.0L

PDA Medium

Difco Potato Dextrose Agar	39.0g
MilliQ Water	to 1.0L

S1M Medium

NH_4Cl	3g
$MgSO_4 \cdot 7H_2O$	2g
$FeSO_4 \cdot 7H_2O$	0.2g
KH_2PO_4	2g
Peptone	2g
Yeast Extract	2g
Malt Extract	2g
Glucose	20g
MilliQ Water	to 1.0L

STC Buffer

Sorbitol	1.2M
Tris-HCl, pH 8.0	10mM
$CaCl_2$	50mM

Cas-9 Working Buffer

HEPES	20mM
KCl	150mM

Adjust to pH 7.5

PEF(4000)-$CaCl_2$ Buffer

PEG(4000)	60% (w/v)
$CaCl_2$	50Mm
Tris-HCl, pH 7.5	450Mm

Media and Reagent Formulations Used: In WT Secondary Metabolite Profiling
* agar was only included for solid cultures

CS Medium (1L)

Cottonseed Flour	20g
Soy Peptone	2g
Maltose	40g
$MgSO_4 \cdot 7H_2O$	2g
NaCl	2g
$CaCO_3$	3mg
MilliQ Water	to 1.0L
Agar*	20g

CYA Medium (1L)

$NaNO_3$		3g
KH_2PO_4		1g
KCl		0.5g
$MgSO_4 \cdot 7H_2O$		0.5g
$FeSO_4 \cdot 7H_2O$		10mg
Yeast Extract		5g
Sucrose		30g
MilliQ Water		to 1.0L
Trace Element Solution		1000µL
$ZnSO_4 \cdot 7H_2O$	1g	
$CuSO_4 \cdot 5H_2O$	0.5g	
MilliQ Water	100mL	
Agar*		20g

CYS80 Medium (1L)

Sucrose	80g
Corn Meal	50g
Yeast Extract	1g
$MgSO_4 \cdot 7H_2O$	0.5g
MilliQ Water	to 1.0L
Agar*	20g

PDA Medium (1L)

Difco Potato Dextrose Broth	36.0g
MilliQ Water	to 1.0L
Agar*	20g

MEA Medium (1L)

Malt Extract	20g
Peptone	1g
D-glucose	20g
MilliQ Water	to 1.0L
Trace Element Solution	1000uL
Agar*	20g

MMK2 Medium (1L)

Mannitol	40g
Yeast Extract	5g
Murashige & Skoog Salts	4.3g
MilliQ Water	to 1.0L
Agar*	20g

Q6 Medium (1L)

D-Glucose	2.5g
Glycerol	1.g
Cottonseed Flour	5g
MilliQ Water	to 1.0L
Agar*	20g

S2M Medium (1L)

$(NH_4)_2HPO_4$ -OR- Putrescine	1g
KH_2PO_4	3g
$MgSO_4 \cdot 7H_2O$	0.2g
NaCl	5g
Sucrose	40g
Glycerol	10g

NPN-A Medium (1L)

S1M	10%
S2M ($(NH_4)_2HPO_4$)	90%
Agar*	20g

NPN-p Medium (1L)

S1M	10%
S2M (Putrescine)	90%
Agar*	20g

YES Medium (1L)

Yeast Extract	20g
Sucrose	150g
$MgSO_4H_2O$	0.5g
MilliQ Water	to 1.0L
Agar*	20g

YES+IO Medium (1L)

YES Medium	1.0L
Instant Ocean	18g
Agar*	20g

ZM/2 Medium (1L)

Molasses	5g
Oatmeal (Flakes ground)	5g
Sucrose	4g
D-Glucose	1.5g
$CaCO_3$	1.5mg
Edamin	0.5g
$(NH_4)_2SO_4$	0.5g
MilliQ Water	to 1.0L
Agar*	20g

Table S1: List of genes within the C16 BGC with genes used to generate CRISPR-Cas9 KO strains identified in bold.

Gene Cluster	LocusID	Protein Signature
C16	FGSG_04596	O-methyltransferase
	FGSG_04595	Flavin-containing monoxygenase
	FGSG_12222	Terpene cyclase
	FGSG_12223	Short-chain dehydrogenase/reductase 1
	FGSG_04593	Prenyltransferase
	FGSG_04592	Short-chain dehydrogenase/reductase 2
	**FGSG_04591**	**Geranylgeranyl diphosphate synthase (TS)**
	FGSG_04590	Cytochrome p450
	FGSG_04589	O-methyltransferase
	**FGSG_04588**	**Nonreducing polyketide synthase (PKS15)**

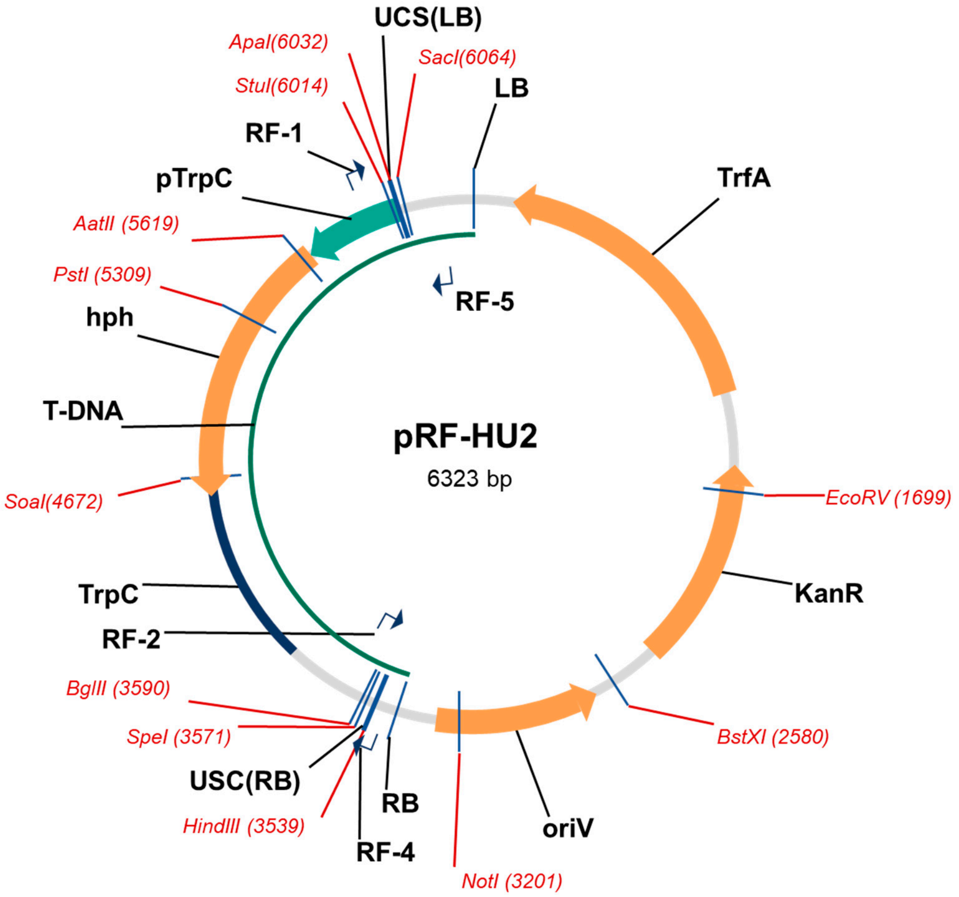

Figure S1: pRF-HU2 vector map used in construction of Hygromycin repair templates

Sequence used for the hygromycin repair template amplified from pRF-HU2 vector. pTrpC promotor sequence is indicated in blue and hygromycin B is indicated in red. Upstream and downstream primers were amplified with regions indicated in green and yellow respectively and HYG588U and HYG588L positive control amplification primers are indicated highlighted in grey.

ctattcctttgccctcggacgagtgctggggcgtcggtttccactatcggcgagtacttctacacagccatcggtccagacggccgcgcttctgcgggcgatttgtgtac
gcccgacagtcccggctccggatcggacgattgcgtcgcatcgaccctgcgcccaagctgcatcatcgaaattgccgtcaaccaagctctgatagagttggtcaagac
caatgcggagcatatacgcccggagccgcggcgatcctgcaagctccggatgcctccgctcgaagtagcgcgtctgctgctccatacaagccaaccacggcctccag
aagaagatgttggcgacctcgtattgggaatccccgaacatcgcctcgctccagtcaatgaccgctgttatgcggccattgtccgtcaggacattgttggagccgaaat
ccgcgtgcacgaggtgccggacttcggggcagtcctcggcccaaagcatcagctcatcgagagcctgcgcgacggacgcactgacggtgtcgtccatcacagtttgcc
agtgatacacatggggatcagcaatcgcgcatatgaaatcacgccatgtagtgtattgaccgattccttgcggtccgaatgggccgaacccgctcgtctggctaagatc
ggccgcagcgatcgcatccatggcctccgcgaccggctgcagaacagcgggcagttcggtttcaggcaggtcttgcaacgtgacaccctgtgcacggcgggagatgc
aataggtcaggctctcgctgaattccccaatgtcaagcacttccggaatcgggagcgcggccgatgcaaagtgccgataaacataacgatctttgtagaaaccatcgg
cgcagctatttacccgcaggacatatccacgccctcctacatcgaagctgaaagcacgagattcttcgccctccgagagctgcatcaggtcggagacgctgtcgaact
tttcgatcagaaacttctcgacagacgtcgcggtgagttcaggctttttcatatcgatgcttgggtagaataggtaagtcagattgaatctgaaataaagggaggaagg
gcgaacttaagaaggtatgaccgggtcgttcacttaccttgcttgacaaacgcaccaagttatcgtgcaccaagcagcagatgataatatgtcctcgttcctgtctgct
aataagagtcacacttcgagcgccgccgctactgctacaagtggggctgatctgaccagttgcctaaatgaaccatcttgtcaaacgacacaaattttgtgctcaccgc
ctggacgactaaaccaaaataggcattcattgttgacctccactagctccagccaagcccaaaaagtgctccttcaatatcatcttctgtcga

Table S2: Primers used in the amplification of repair templates

Gene Cluster	Primer ID	Location	Primer Name	Nt	Primer Sequence 5'-3'
FGSG_04588	5	DO	FGSG_04588hygMicroDO	70	CACCACAATTAATCCCGTCAGGTTACAGCTCTCTGTGGTCTAGCTAAACGCTATTCCTTTGCCCTCGGAC
		UP	FGSG_04588hygMicroUP	75	CCAAATACGAATAATATAGTAAAAGAATGTGATTAGTCTGCATTGCTTCCGTCGATCGACAGAAGATGATA TTGA
FGSG_04591	6	DO	FGSG_04591hygMicroDO	70	CGTCCGATGATGGGTCCGTAGAACAGCAGCTACTCAGGGTGTCTCTTTATCTATTCCTTTGCCCTCGGAC
		UP	FGSG_04591hygMicroUP	71	GAGACCGTTTAGTTGACATATCTTGTATATTCTAATAGTGGTGTCTTATCGTCGACAGAAGATGATATTGA

Table S3: List of crRNA primers

Gene	crRNA Name	Length	Sequence
FGSG_04588	04588gRNA-down1	36	GAACGGGAUACCAGUUCUGGGUUUUAGAGCUAUGCU
	04588gRNA-up1	36	AUUAGUCUGCAUUGCUUCCGGUUUUAGAGCUAUGCU
FGSG_04591	04591gRNA-down1	36	GGAUGGAACCGUGUGACUCUGUUUUAGAGCUAUGCU
	04591gRNA-up1	36	GUAAUAGUGGUGUCUUAUCGGUUUUAGAGCUAUGCU

Table S4: List of GOI internal primers used for confirmation of gene knockout

Gene	CH Primer ID	Primer Type	Direction	Primer Name	Nt Count	Tm (°C)	Primer Sequence 5' - 3'	Amplicon Size (bp)
FGSG_04588	5	Int	F1	FGSG_04588IntF1	20	56.9	CCATCAGTACGTCCGCTCAA	442
		Int	R1	FGSG_04588IntR1	20	57.1	CTCTGGGCCATATCCGTCAC	
FGSG_04591	6	Int	F1	FGSG_04591IntF1	20	57.3	TGGGCATGATTGGACTGGAG	350
		Int	R1	FGSG_04591IntR1	20	57.7	GTATGACCTGAGCTGCTGGG	

Table S5: Refer to SI_Table_5.xlsx

Table S6: Refer to SI_Table_6.xlsx

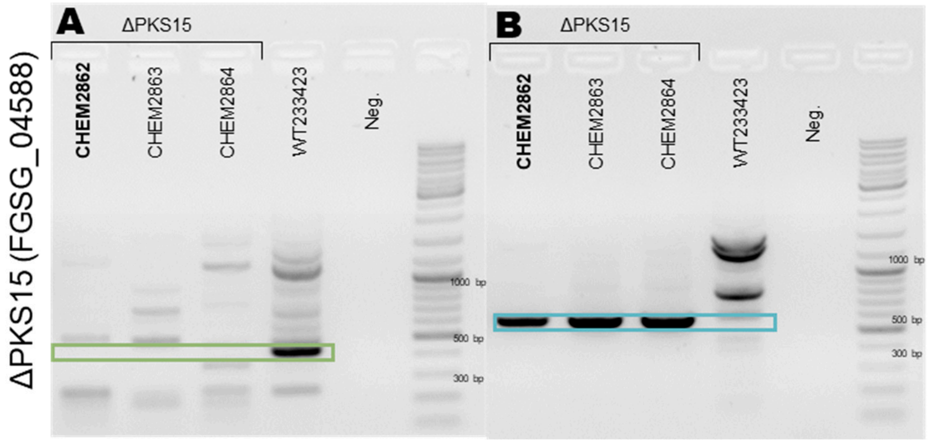

Figure S2: PCR validation of FGSG_04588 KO strains by A) absence of amplification of the GOI as determined using primers internal to the GOI and B) presence of the hygromycin gene. Bolded strain indicates the strain used phenotype analysis and southern blot analysis.

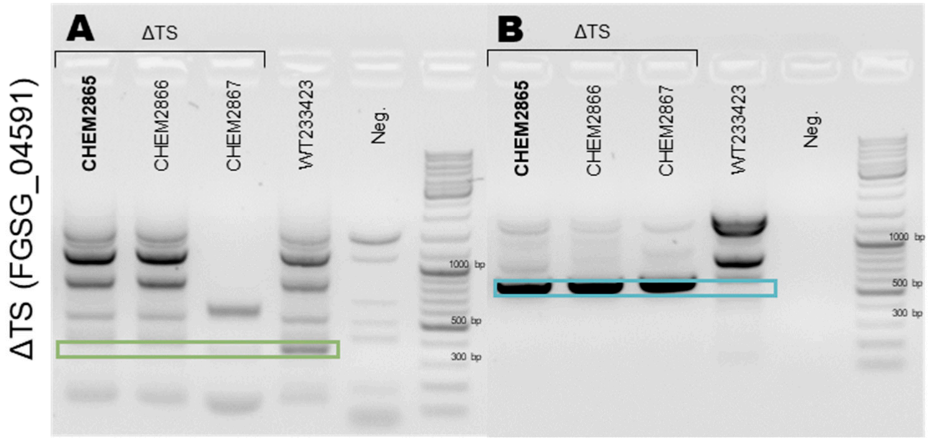

Figure S3: PCR validation of FGSG_04591 KO strains by A) absence of amplification of the GOI as determined using primers internal to the GOI and B) presence of the hygromycin gene. Bolded strain indicates the strain used phenotype analysis and southern blot analysis.

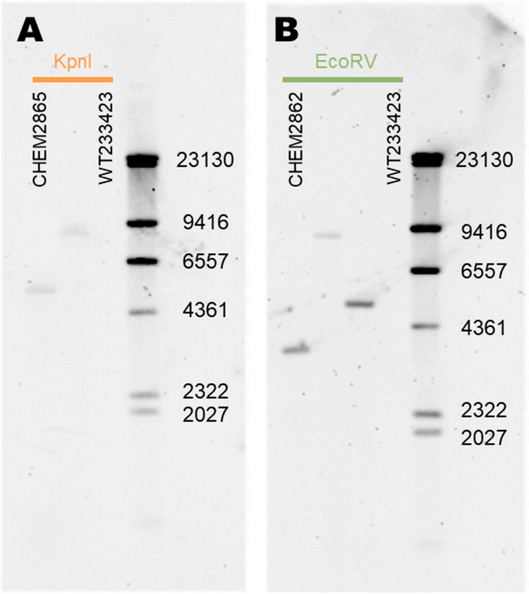

C

Gene Cluster	Strain	Gene KO	Enzyme	Expected Fragment Size (bp)
C16	CHEM2865	FGSG_04591; ΔTS	KpnI	5208
-	WT233423	-	KpnI	X
C16	CHEM2862	FGSG_04588; ΔPKS15	EcoRV	3627
-	WT233423	-	EcoRV	X

Figure S4: A) & B) Southern blot analysis of select transformants with hybridization probe specific of the *hygB* gene. Strains were organized by the enzyme used in the gDNA digest A BamHI and KpnI and B SacI and EcoRV. C) expected sized of the bands for each of the knockout strains.

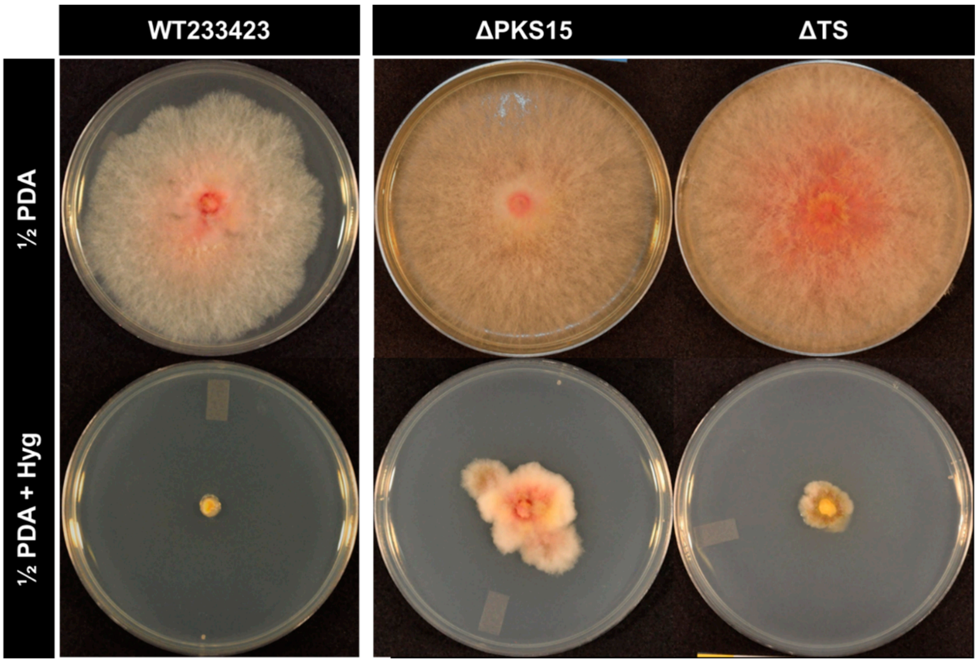

Figure S5: Phenotypes of respective *F. graminearum* WT and transformant strains cultured on ½ PDA (top) and ½ PDA supplemented with 100mg/L hygromycin (bottom).

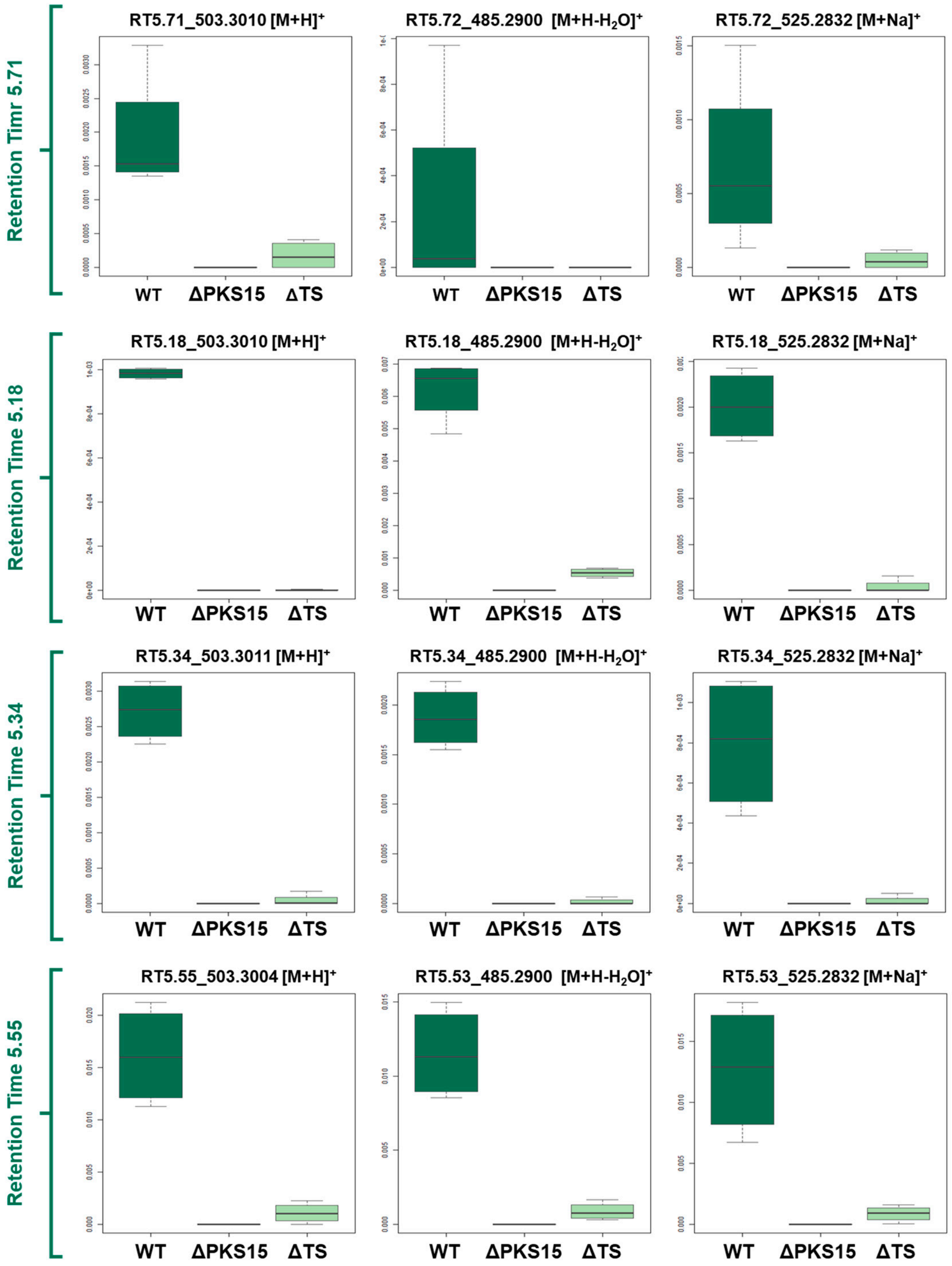

Figure S6: Observed *in planta* production of FDDP molecules by *F. graminearum* WT and transformant strains observed from UPLC-HRMS profiles of infected wheat head extracts.

Table S7: MS2 fragmentation comparison between values reported by Seidl et al, (2022) for gramiketide A and corresponding values observed from unknown mass feature RT5.51_*m/z* 503.3004 in this study.

	Seidl et al, (2022) for gramiketide A	Unknown mass feature RT5.51_*m/z* 503.3004	Mass Error (ppm)
Precursor	503.3003	503.3003	
Fragment 1	485.2891	485.2865	Δ5.357
Fragment 2	211.0596	211.059	Δ2.843
Fragment 3	197.044	197.0433	Δ3.553
Fragment 4	183.0284	183.0276	Δ4.371
Fragment 5	179.0335	179.0329	Δ3.351
Fragment 6	131.0337	131.0332	Δ3.816
Fragment 7	113.0233	113.0226	Δ6.193

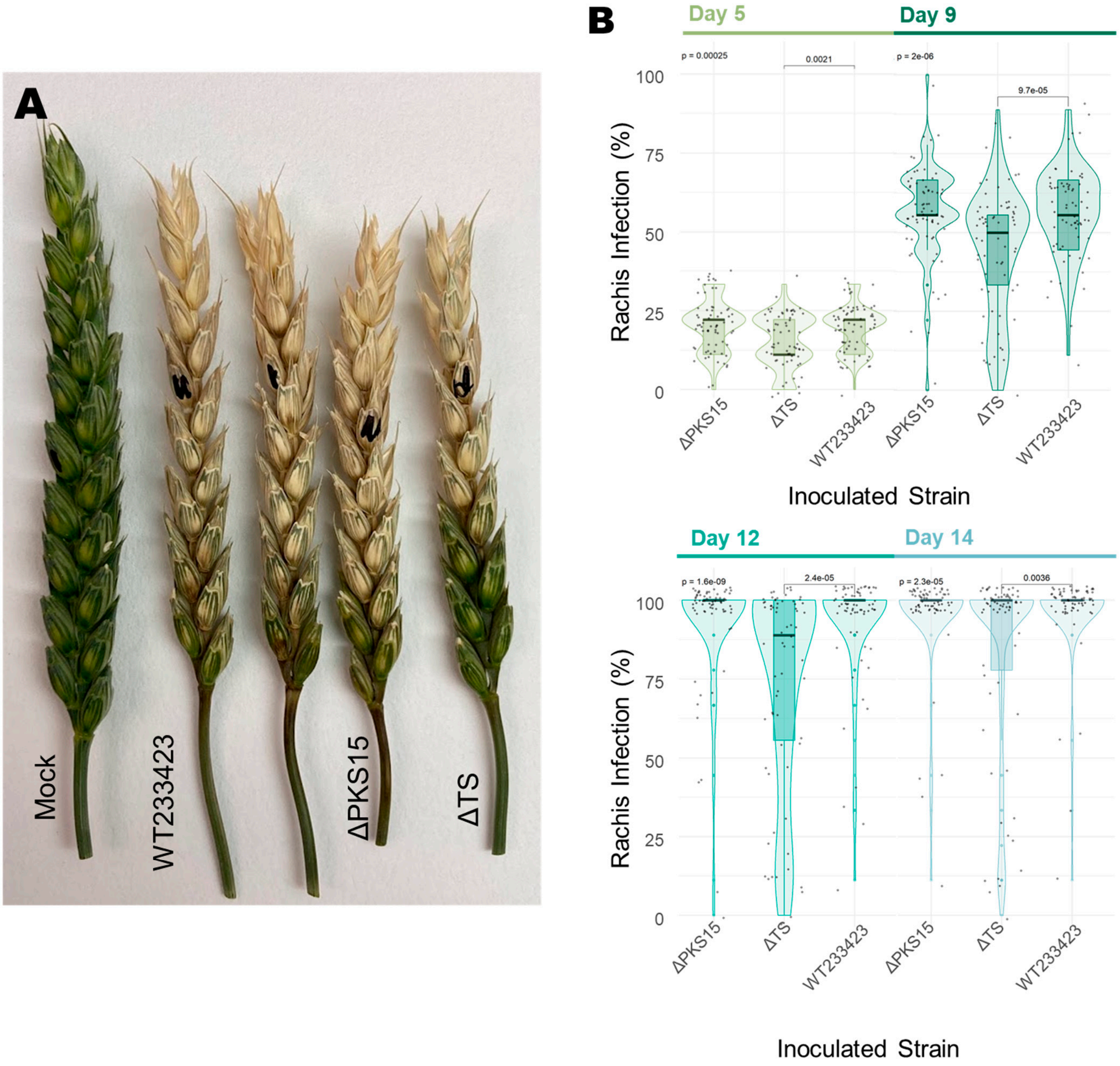

Figure S7: A) Observed phenotype differences 14 days post inoculation from wheat head challenge with *F. graminearum* WT and transformant strains (ΔPKS15 and ΔTS) as compared to mock infection control. Spikelets used for as point of inoculation are designated with black stripe. B) Violin plots tracking the % rachis infection rate 5, 9, 12 and 14 days post infection.
